# New Graduate Transition Programs: It’s All About Engagement

**DOI:** 10.3390/nursrep16070211

**Published:** 2026-06-23

**Authors:** Marcia A. Docherty, Andrea L. Taylor, Josh T. Duncan

**Affiliations:** 1 School of Leadership Studies, Royal Roads University, Victoria, BC V9B 5Y2, Canada; 2 Professional Practice & Learning, Island Health, Victoria, BC V8R 1J9, Canada

**Keywords:** new graduate transition to practice, new nurses, program evaluation, action research, engagement, nursing

## Abstract

**Background/Objective:** Island Health has offered a New Graduate Transition Program (NGTP) since 2005 and refreshed its existing curricular program for the 2022 to 2024 new nurse graduates as the BC healthcare system entered pandemic recovery. **Methods:** An engaged, action-oriented evaluation strategy, using iterative cycles of Look—Think—Act, was used to inform the program refresh and build relationships with user groups and key interest holders. **Results:** In 2021, engagement of new graduates in the NGTP ranged from 0.01 to 36%. The refreshed program increased their engagement between 14% and 61%. New graduates leaving Island Health after one year in practice decreased from 7% in 2021 to 4% in 2023. New graduates occupying casual positions decreased from 24% in 2023 to 8% in 2024. **Conclusions:** This paper underscores the critical importance of intentional relationship building as a foundational element in ensuring new nurse graduates are not only welcomed but successfully integrated into their professional roles with confidence and competence.

## 1. Background

New nurse graduates often experience difficulties transitioning from their student role into their professional role [1,2,3]. Duchscher’s transition theory maps three stages a new nurse transitions through during their first year in practice: resolving the shock of full nursing practice, followed by resolving the self-doubt of choosing nursing as a profession, and finally creating an identity as a confident and competent nurse [4]. This initial and often unstable year had only been made more challenging by the COVID-19 pandemic [5,6]. Despite these difficulties, new nurses are committed to excellence and expect to be supported in their unique career goals by their peers and their organization [7,8,9]. Therefore, Masso et al. advocate for a shift in transition programs, from focusing solely on nurse readiness to emphasizing workplace readiness [10]. This approach recognizes that successful integration of new graduates depends not only on individual preparedness, but also on the capacity of the work environment to support, mentor, and retain them. Supportive work environments that include mentorship [11,12] and standardized transition programs [13,14] can be effective for assisting the new nurses’ consolidation of theory with clinical skills and improve retention [4,15,16,17]. However, further evaluation of these programs is warranted [1,18].

Since 2005, Island Health has offered a formal New Graduate Transition Program (NGTP) that included core elements of employment opportunities, supernumerary time, education, and clinical mentorship practice supports. The goals of Island Health’s NGTP remain consistent: to support new nurses in consolidating theory into practice, build their capacity to navigate the challenges of their first year, and provide the resources and tools needed to support standards of excellence in entry-level, professional practice. Ideally, the program enhances the confidence, competence, and connections of new graduates. While the NGTP is inclusive of all health professions, its activities have primarily focused on nursing graduates; this emphasis reflects both the volume of new nurse hires and the unique challenges they face during their transition to practice. However, the degree to which these elements were implemented has varied year over year, and ensuring engagement of new graduates has been challenging. For example, in 2021, a town hall was provided along with nine virtual cafes and an online, self-directed course. Attendance at the town hall was 36%, while engagement in the virtual cafes and self-directed course was 0.01%.

In 2022, as the BC healthcare system entered pandemic recovery, BC health authorities, with support from the BC Ministry of Health’s Health Human Resource initiatives, refreshed and strengthened NGTPs in recognition of the impacts on undergraduate and pre-licensure education experiences during the pandemic, the current health human resource context of limited resources, a pressured practice environment, and concern for the health and wellness of nurses [19]. In response, Island Health developed three in-person new graduate transition sessions using an engaged, action-oriented approach to promote new graduate, educator, mentor, and leader engagement in the sessions.

### Island Health’s New Nurse Graduate Transition Framework and Program

Each spring, Island Health hires over 200 new RN and RPN graduates. In 2022, Island Health’s refreshed NGTP included protected time for transition activities of clinical buddy shifts, a minimum of 144 h of protected orientation time in addition to other orientation activities, and 30 h of paid transition support and new graduate transition professional development (PD) sessions. To support these activities, Island Health also launched a Clinical Mentorship pilot, which included the hiring of five clinical nurse mentors across different sites. These formal mentors provided regional, new graduate support and participated in the design and delivery of the PD sessions alongside other educators and new graduate nurse representatives. Professional Practice has operational oversight for these initiatives and designated one New Entrant Navigator and one New Entrant Lead to provide enhanced support to new graduates, operational managers, leaders, educators, and clinical mentors. The New Entrant Navigator led the scheduling and delivery of the PD sessions and the New Entrant Lead designed, developed, and evaluated the PD sessions.

The PD sessions were designed using an engaged, action-oriented approach. Action research is a collaborative approach to quality improvement that leverages reflection on shared experiences to co-create knowledge through iterative cycles of Look–Think–Act [20,21]. Data was collected (look) and analyzed (think) by a working group prior to, during, and post implementation from a range of key sources, including the user group, and informed the iterative development (act) of the PD sessions over the period of a year through 5 cycles of Look—Think—Act (see Table 1).

An evaluation plan was developed that provided formative feedback for the next PD session and summative feedback for future NGTP activities. This paper presents the formative and summative evaluation findings from the 2022, 2023, and 2024 offerings of the NGTP.

## 2. NGTP Evaluation Method

Island Health’s NGTP begins in May, which corresponds to the graduation date of local nursing programs. In the first iterative cycle, topics were informed by Duchscher’s new graduate transition theory [4] and generated from an analysis of the 2021 PD sessions and activities, a review of professional practice consultations by a working group of educators, and the 2022 cohort were invited to complete a new graduate survey: *What Matters to You?*, administered three months post hire. PD sessions were held in their fifth, ninth, and 13th months of their transition, and attendees were asked to evaluate each session and provide ideas for future topics. Presenters from the PD sessions were also brought together approximately two weeks post session to debrief the session. They provided feedback on what went well, what could be improved, and anything learned from the new graduates or others. We also obtained feedback from new graduate representatives who attended the session. All feedback was themed and used to inform the next PD session. In addition to the session evaluations, all new graduates were then invited to evaluate the total NGTP 13 months post hire, regardless of whether they attended any PD sessions or not (see Table 1).

From the 2022 data, a program of topics was finalized for the 2023 cohort (see Table 2).

Based on the feedback, the PD sessions were held earlier, in the second, fifth, and seventh months. New evaluations were developed for the 2023 PD sessions that shifted focus away from identifying learning topics and instead evaluated the application of the session’s learning to practice. These evaluations were again collected after each PD session. An evaluation of the total NGTP was administered one month after the third PD session, at eight months. In 2024, based on feedback from 2023, the PD sessions were again held in the second and fifth months post hire and the third session was not offered due to the PD sessions undergoing further re-evaluation and revision. An evaluation of the total NGTP was administered at the one-year mark, in May 2025 (see Table 3).

Session and total program evaluations were collected and anonymized in REDCap. Quantitative Version 16.0.22 data was analyzed in REDCap and qualitative data was manually themed using Excel (Microsoft Corporation, Redmond, WA, USA, 2021). This project is registered with Island Health’s QI Registry.

### 2.1. Findings

The evaluation strategy had two goals. The first was to inform the quality of the PD sessions, which included identifying topics and satisfaction with the sessions. The second was to inform the quality of the refreshed NGTP, which included not only information on the PD sessions but also information on protected orientation supports.

### 2.2. Professional Development Sessions

#### 2.2.1. Attendance

In 2021, topic-specific sessions were offered virtually throughout the year with very little engagement. Therefore, full-day, in-person sessions were piloted in 2022, which showed a significant increase in participation by new graduates. Engagement activities with the new graduates and their leaders, educators, and recruiters about the refreshed NGTP was instrumental in increasing awareness. The recruitment team provided a contact list of all the new graduates and their leaders that the New Entrant Navigator used to circulate invitations to the PD sessions, and a dedicated email account was created to improve communication. Additionally, the New Entrant Lead presented the details of the refreshed program at various meetings and Town Halls. Attendance increased significantly with the refreshed NGTP. However, as the transition year progressed, attendance dropped (see Table 4).

The primary reasons identified for not attending were: (1) Not interested (38%), (2) not released from shift (21%), (3) wasn’t aware of it (14%), (4) unit orientation was sufficient (9%), (5) on a leave (9%), (6) notifications weren’t timely (5%), or (7) Other (4%) which included not wanting to travel to the session and choosing to pick up an additional shift instead (*n* = 70).

Based on feedback and attendance in 2022, the 2023 PD sessions were hosted earlier in the transition period. This did support slightly improved attendance for Sessions 2 and 3; however, the attendance for Session 3 remained low. Therefore, in 2024, we elected to pause the third session to determine if there was an impact.

#### 2.2.2. Learning

In 2022, the session evaluations focused on identifying topics the new graduates wanted to learn about. The following topics were identified most frequently by respondents (*n* = 238):

1. New Graduate Transition to Practice.
2. In-Charge.
3. Ethical Decision Making.
4. Prioritizing your Day.
5. Medication Safety.
6. Networking with other New Graduates.

Respondents were also asked for suggestions on how to improve these sessions. Responses (*n* = 82) were themed into three categories of improvements for session delivery (35%), improvements for PD content (33%), and improvements on how the sessions were organized (20%).

In 2023, with the topics finalized, the evaluation was changed and respondents were asked how they will apply what they learned in the sessions. Responses from 2023 and 2024 (*n* = 189) were grouped into five categories based on frequency.

From their responses, the thematic analysis identified that the sessions supported:

- Navigation of the clinical system, making connections, and identifying resources (38%).
- Self-directed learning and career planning (24%).
- Skills and confidence for self-advocacy (16%).
- Locating the source of truth for human resources and union processes and practices (11%).
- An overall feeling of being supported and appreciated (10%).

#### 2.2.3. Satisfaction

Satisfaction with the session was measured through an evaluation that focused on competence and confidence (see Table 5). Overall, satisfaction with the session remained consistently high in 2022 and 2023 with a drop in satisfaction in the 2024 sessions.

These results may indicate that changes in the undergraduate curriculum at the post-secondary and widespread implementation of the clinical nurse mentor role have had positive effects on the transition experience and that the session content, which was created to support new graduates impacted by the pandemic, may no longer be relevant for post-pandemic cohorts. Therefore, Professional Practice decided not to offer a third session in 2024, with plans to re-evaluate and revise the current PD curriculum to meet the emerging needs of new nurses.

### 2.3. Overall Feedback on the NGTP

#### 2.3.1. Professional Development Sessions

In addition to evaluating the PD sessions, a survey was sent out to all new graduates hired to gather feedback on the entire NGTP, including PD sessions and protected orientation supports. The 2022 new graduates evaluated the NGTP 13 months post hire, the 2023 new graduates evaluated the NGTP eight months post hire, and the 2024 new graduates evaluated the NGTP 12 months post hire (see Table 3). The 2023 survey was administered within what Duchscher describes as the “transition crisis phase”, a time marked by emotional, cognitive, and professional upheaval as new graduates adjust to the realities of practice [4]. Interestingly, almost twice as many respondents in the 2023 survey identified that they were considering leaving the nursing profession from the 2022 survey respondents [4]. It was unclear whether these responses validated Duchscher’s transition model [4] or reflected issues with Island Health’s NGTP. Therefore, we recommend evaluating the program at 12 months when new graduates have completed the recovery and consolidation stage of the transition experience.

Respondents (*n* = 86) consistently appreciated the designated time to come together as a new graduate community to share experiences and feelings, ask questions, and learn about Island Health clinical resources as well as the human resource practices and policies not taught in the post-secondary institutes. The quality of support from the New Entrant Navigator and the personalized and standardized learning approaches within the sessions were also appreciated:

*I appreciated having the space during the three sessions within the first six months to discuss challenges and have questions answered by [the New Entrant Navigator] and other Island Health staff who came to the sessions.*

*(2023 NG)*

*[The PD Sessions were] helpful in some ways. The development sessions felt similar to what we learned in school but the hands-on and practical learning was beneficial (i.e., panel of professionals—BCNU, HR, CNE; in-charge).*

*(2022 NG)*

Most of the feedback on improvement focused on improving the relevance of the sessions for all attendees, specifically those attendees who were working in non-acute care areas such as community and mental health and substance use. More information about benefits, shift scheduling, and the union continues to be requested. New graduate attendees suggested improving the delivery of the PD sessions by increasing the diversity of facilitators/panel members and improving the scheduling so that new graduates not hired in May and those new graduates who are not able to attend can participate. Further engagement to ensure department support in attending is warranted. Finally, several respondents expressed a desire for more support (mentors, check-ins, manager engagement) during their protected orientation time.

#### 2.3.2. Protected Consolidation Time

During their first year of practice, new graduates thrive in stable, supported, and predictable environments [4]. Ideally, this period focuses on consolidation of entry-level skills and nursing practice, and they should be protected from advanced practice activities, such as being in charge or deployed to other departments. However, protecting this time is challenging, particularly during the summer months, when experienced nurses are on vacation and the unit is relying on agency nurses to maintain full staffing. Half of the respondents (49%) had been assigned in-charge shifts, with most of these shifts occurring within the first three months of employment. Less than half of respondents (43%) were deployed during the first six months, with the majority being deployed one to three times.

## 3. Discussion

The in-person sessions were much more successful than the virtual and self-directed sessions offered in 2021. The timing of the PD sessions necessitates further review. Offering PD sessions multiple times throughout the year may better support new graduates who are hired outside of the new graduate hiring window. Scheduling the in-person PD sessions within the first and fifth month seemed to best support the new graduates’ initial transition needs and allow for positive relationship building. To further enhance the transition experience, identifying an effective third in-person session scheduled closer to the end of the transition period is recommended. The goal of this session would be to reinforce learning and provide continued support as the new nurses progress in their role. Conversely, we have considered combining the first and third in-person sessions to foster mentorship opportunities between senior and junior new graduates.

The PD sessions may have contributed to improved new graduate retention as they provided a forum for new graduates to learn how to navigate their career more effectively. New graduates leaving Island Health after one year in practice decreased from 7% in 2021 to 4% in 2023. During this same time frame, there was a corresponding increase in new graduates changing positions from 3% to 7% and we saw a reduction in new graduates occupying casual positions decrease from 24% in 2023 to 8% in 2024.

The need to balance practical information while being inclusive of all care areas continues to be challenging. With the focus of new graduate transitions expanding to LPNs and allied health, there is an opportunity to explore how all new graduates could be transitioned together to support strategic practice goals such as collaborative care, cultural humility, and governance.

Having access to mentors was critical to new graduates. They frequently called on site-specific mentors for immediate clinical support and reached out with questions and concerns to the New Entrant Navigator. The Navigator and PD sessions were often the first direct contact new graduates had with Professional Practice, widening their perspectives of professional resources and processes within Island Health. Fully integrating clinical mentor and Navigator positions into the NGTP is important to ensure coordination and relevance of the NGTP. With these roles, it was possible to create a shared space to talk about the actual experiences of nursing within a complex healthcare system that resulted in meaningful relationships with and between the new graduates. An opportunity also existed to identify transition gaps in clinical skills and knowledge and provide this information back to post-secondary institutes to collaboratively reduce the gap between supervised and independent practice.

The longitudinal engagement with clinical mentors and educators in this project improved the alignment between hospital/department orientations and the PD content to reduce overlap, improve relevance, and increase NGTP attendance. Anecdotal feedback from clinical educators is that the new graduates are better colleagues when they attend the PD sessions. Engaging leaders in the NGTP was more challenging and regional gaps in hospital/department orientations continue to exist. The development of a coordinated, regional NGTP that is evidence-informed on topics such as medication safety, time management, and documentation practices is recommended.

While many respondents mentioned the importance of developing a competent nursing practice, very few respondents specifically linked this learning to supporting quality patient care. This suggests that the PD sessions may need to focus more on patient advocacy and quality patient care by encouraging nurses to use their voices effectively to enact positive health system reform. This suggests the PD sessions may need to directly center the patient in the new graduate’s transition to practice and leverage patient safety data to connect practice with quality patient outcomes.

Finally, developing an engaged, action-oriented evaluation strategy to support the NGTP provided rich data and provided a mechanism for relationship building. The gathering and sharing of data facilitated increased awareness of the program across the organization and contributed to quality improvement of the program and improvement to local and other regional new graduate orientation activities.

## 4. Limitations

Collecting evaluation data to support engaged, action-oriented improvement resulted in the data collection tools and timelines evolving, making it challenging to compare data across the cohorts. However, the benefits of responding to the evaluation data in a timely manner improved engagement in the evaluation strategy.

Including a control group of new graduates who were not enrolled in the transition program and longitudinal data collection was not built into the design of this quality improvement initiative. Therefore, it is unclear from this study whether the improvements to satisfaction and perceived supports were attributed directly to the transition program or the result of other factors such as specific unit culture, educational supports, or staffing composition, and we are uncertain about the long-term impacts of the program.

## 5. Conclusions

While it is not always possible to fully ease the transition to practice, this research suggests that an NGTP with protected orientation and learning time as well as relationship building across the organization made a meaningful contribution to the new graduates’ success. Furthermore, a key benefit of the NGTP is its strong emphasis on professional development early in a nurse’s career. In-person professional development sessions were central to creating a safe space for the new graduates to gather with their mentors, share experiences, build peer relationships, and align their professional nursing practice with the values and expectations of the healthcare organization.

## Figures and Tables

**Table 1:** Action research involves iterative cycles of Look–Think Act [20,21].

Cycle 1	Cycle 2	Cycle 3	Cycle 4	Cycle 5
**Look** Analysis of 2021 New Graduate PD Sessions & Activities Pre-Program Survey: *What Matters to You*? Review of Professional Practice Consults	**Look** Session 1 Evaluation Session 1 Facilitator Debrief Undergraduate Nursing Students Debrief	**Look** Session 2 Evaluations Session 2 Facilitator Debrief	**Look** Session 3 Evaluation Session 3 Facilitator Debrief	**Look** Total Program Survey New Graduate Nurse Workforce Retention Data
**Think** With the working group of educators, practice consultants, nurse mentors, and new graduate nurse representatives.				
PD Session 1 Content Development	PD Session 1 Improvements PD Session 2 Content Development	PD Session 2 Improvements PD Session 3 Content Development	PD Session 3 Improvements	Year 2 PD Session Improvements
**Act** PD Session 1 Hosted	**Act** PD Session 2 Hosted	**Act** PD Session 3 Hosted		**Act** Year 2 PD Sessions

**Table 2:** 2023 professional development session topics.

2023 Professional Development Session Topics		
Session 1	Session 2	Session 3
Psychological Safety New Grad Transition to Practice Prioritizing Your Day In-Charge Panel: Human Resources, Educator, New Graduate Learning Plans	Interprofessional Collaboration Medication Safety Conflict Resolution Time Management Documentation Professional Responsibility Process (Nurses Bargaining Association) Panel: Professional Practice, Human Resources, Union, Manager	Nursing Leadership and Advocacy Cultural Safety and Humility Ethics in Nursing Quality Improvement Learning Plans

**Table 3:** Evaluation timeline and participation.

Evaluations	2022			2023			2024		
	Timeline	Post Hire (Months)	*n*	Timeline	Post Hire (Months)	*n*	Timeline	Post Hire (Months)	*n*
**What Matters to You Survey**	August	4	87						
**Session 1 Evaluation**	September	5	94	June	2	115	June	2	105
**Session 1 Facilitator Debrief**	November		7						
**Session 2 Evaluation**	January–February	8	53	September	5	54	September	5	43
**Session 2 Facilitator Debrief**	February		4						
**Session 3 Evaluation**	May	13	21	November	7	40			
**Session 3 Facilitator Debrief**	June		4						
**Total NGTP Evaluation**	June–August	14–16	32	December	8	38	May 2025	12	16
**Total**			**302**			**247**			**164**

**Table 4:** Attendance of new graduates at the professional development sessions.

NGTP Historical Attendance		NGTP Refresh Attendance			
	2021		2022	2023	2024
**Town Hall (1)**	36%	**In-Person Session 1**	61%	56%	59%
**Virtual Cafes (9)**	0.01%	**In-Person Session 2**	38%	45%	30%
**Online Course**	0.01%	**In-Person Session 3**	14%	29%	NA

**Table 5:** Evaluation results of all sessions.

Questions	Agree/Strongly Agree		
	2022 (*n* = 168)	2023 (*n* = 209)	2024 (*n* = 149)
I felt welcome to share my ideas during today’s session.	96%	97%	95%
I was provided enough opportunity to practice the competencies covered in today’s session.	79%	78%	72%
I will continue to connect with my new graduate peers after today’s session.	73%	86%	87%
Today’s session supported me in the journey to consolidate my practice.	81%	90%	78%
Today’s session supports my ability to apply theory to practice.	77%	83%	70%
Having completed this workshop, I feel more prepared to work through the transitions that may occur during my first year of practice.	75%	84%	70%
Having completed this workshop, I feel more confident on how to access resources and tools to support me in my professional development.	85%	92%	82%
Overall, I am satisfied with today’s session	--	90%	78%

## Data Availability

Data available on request due to privacy restrictions.

## Public Involvement Statement

No public involvement in any aspect of this research.

## Guidelines and Standards Statement

This manuscript was drafted against the Standards for Reporting Qualitative Research, O’Brien B, Harris IB, Beckman TJ, Reed DA, Cook DA. Standards for reporting research: A synthesis of recommendations. *Acad Med.* 2014;89:1245–1251. https://doi.org/10.1097/ACM.0000000000000388 (qualitative research).
